# Challenges and health outcomes of the exposure to soybean dust in the harbor neighborhood of Karachi, Pakistan: a wake-up call

**DOI:** 10.1186/s41043-023-00473-4

**Published:** 2023-11-30

**Authors:** Nadeem Ullah Khan, Asrar Ali, Umerdad Khudadad, Uzma Rahim Khan, Noman Ali, Salman Muhammad Soomar, Shehryar Abid, Mahmood Jilani, Seemin Jamali, Junaid A. Razzak

**Affiliations:** 1 Department of Emergency Medicine, Aga Khan University, Karachi, 74800 Pakistan; 2 Ziauddin University Hospital, Karachi, Pakistan; 3 Jinnah Postgraduate Medical Center, Karachi, Pakistan; 4 Weil Cornell Medicine, New York, USA

**Keywords:** Chemicals, Toxicology, Hazard, Exposure, Health

## Abstract

**Background:**

Chemical disasters are common worldwide and result from technological failure, war, and terrorism activities. Pakistan imports huge quantities of hazardous chemicals to meet its industrial and energy needs. Hence there is a risk of chemical disaster at the ports, during transportation of such material and processing in the chemical industry. This study aimed to review the challenges and health outcomes of cases of soybean dust exposure in Kemari district (harbor neighborhood) of Karachi, Pakistan.

**Methods:**

A cross-sectional survey was conducted with all the affected people from a chemical incident of soybean dust which was reported in the Keamari district of Karachi, Pakistan. Included patients ≥ 18 years who visited the two major tertiary care hospitals of Karachi, Pakistan after the incident between February 17 to 23, 2020. A total of 574 patients were brought to these two major tertiary care hospitals. We collected data on basic demographics, event details, and major signs and symptoms of the affected individuals. Calculated frequencies and percentages for categorical variables. Mean ± standard deviation (SD) was calculated for continuous variables.

**Results:**

The mean ± (SD) age of the victims were 32 (13.5) years. Of the 574 patients, majority of the patients (*n* = 319, 56%) were males. In 28 cases (41%), the onset of symptoms occurred at home, in 27 cases (39%) the onset of symptoms started in the workplace and the remaining cases (*n* = 14, 20%) experienced the first symptoms while roaming around the roadside. The most common reported co-morbidity was a history of asthma (56%), followed by diabetes mellitus (22%). The most common clinical manifestation was shortness of breath, reported in 94% of the cases, followed by neurological symptoms such as drowsiness, unconsciousness, or seizures experienced by 10% of the victims. A total of 9 deaths (1.5%) were recorded.

**Conclusion:**

A multi-sectoral systematic approach is also required to address these incidents comprehensively including the trained and equipped pre-hospital system, integrated emergency medical response, and community-wide emergency response system.

## Introduction

A chemical incident is an unexpected release of an industrial chemical that is potentially hazardous to humans, animals, or the environment [1]. Chemical disasters are common worldwide and result from technological failure, natural disasters, war, and terrorism activities [2]. In the United States, 60,000 chemical spills, leaks, and explosions involving more than 300 deaths occur each year [3]. Many incidents of the chemical disaster in developed countries have been narrated in the literature since World War I. The catastrophic incident of the rupture of the ammonia gas pipeline affected nearly 75 people in London, out of which 7 died [4]. In addition, leakage of phosgene gas in Hamburg, Germany affected around 300 natives and caused mild to moderate respiratory complications; out of which 10 died [5]. Similarly, the number of mortalities related to chemical emergencies has been reported across different countries of the developed region including Australia, Canada, and Japan [6]. Among many chemical agents that tend to cause chemical emergencies; soybean dust has been widely reported in the literature [7–9]. Soybean-induced allergic symptoms can range from skin, gastrointestinal, or respiratory tract reactions up to anaphylaxis [10, 11].

The outbreak of soybean dust allergy was first reported in the city of Barcelona, Spain. However, it was persistent in nature, and from 1981 to 1987, nearly 26 outbreaks of asthma due to soybean dust occurred with over 1000 emergency room admissions. Subsequently, similar epidemics of soybean dust allergy have been reported in many countries in the past including the United States, Argentina, and Italy [12–14].

Pakistan imports huge quantities of hazardous chemicals to meet its industrial and energy needs. Hence there is a risk of chemical disaster at the ports, during transportation of such material and processing in the chemical industry [15].

A chemical incident was reported in the Keamari area of Karachi, Pakistan on February 17, 2022, that claimed the lives of 14 persons in 3 days and affected over 500 individuals. There were anecdotal reports of different chemicals involved in this incident including methyl bromide, hydrogen sulfide, nitric oxide, and carbon monoxide [16]. However, the International Center for Chemical and Biological Sciences (ICCBS) investigated the blood samples of affected cases and confirmed that the outbreak incident was due to soybean dust which was unloaded in bulk at the Karachi harbor. An advisory was issued by the health department of Sindh to address the complications of soybean dust allergy across Karachi [17]. This study aimed to review the challenges and health outcomes of cases of the soybean dust outbreak in the Kemari area of Karachi from the perspective of chemical incident management.

## Methods

### Study design

The study design was a cross-sectional survey. We called the affected patients who visited the two major hospitals after the incident to collect data on basic demographics, event details, and major signs and symptoms.

### Study setting

The study was conducted in two tertiary care hospitals in Karachi, Pakistan. The incident took place in the Keamari district of Karachi’s Seaport during offloading of soya bean shipment. Keamari is one of the oldest coastal towns located in the western part of the city which includes an extensive coastline, Karachi port, many beaches, mangroves, and some small islands. Although the victims were taken to 4–5 tertiary-care hospitals in Karachi, the largest number of patients were taken to the nearest hospital, a 120-bedded tertiary-care teaching hospital run by a private medical university as a trust hospital for the local population of Keamari. The second largest group of the patients were taken to a public tertiary care hospital, which is a government-operated hospital with 24 h emergency. The emergency room has a capacity of 60 beds and the daily patient flow is nearly 1500.

### Participants

The study participants were 18 years and above residents of the Keamari district and people working in the location affected by the soybean allergic outbreak.

### Inclusion criteria

All the patients with a history of exposure to the hazardous material (HAZMAT) outbreak in Keamari and who presented to the emergency departments between February 17, 2020 (00:00 h) and February 23, 2020 (23:59 h) were enrolled in the study. In addition, patients who presented with respiratory symptoms including cough, shortness of breath, wheezes, stridor, and conjunctivitis in the two hospitals were also approached.

### Variables of interest

We collected basic demographic variables such as age, gender, occupation, and major medical history of the participants. Moreover, we called the affected people to take a detailed medical history including signs and symptoms of the allergic outbreak and clinical management they received in the hospital. Lastly, clinical outcome variables such as disposition from the emergency department, hospital admission, length of hospital stay, major treatment interventions, and mortality were also captured.

### Data collection procedure

**Fig. 1:**
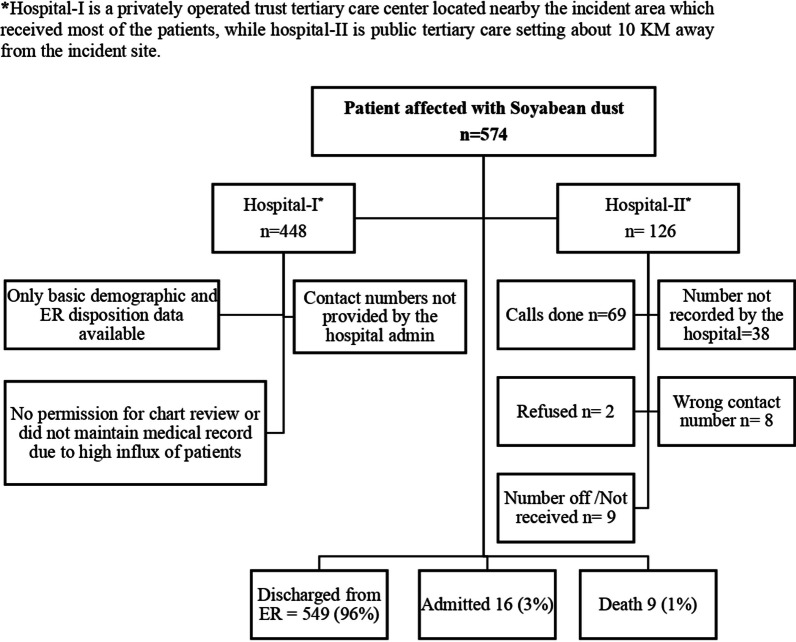
Flow chart showing the details of HAZMAT incidence in Kemari district of Karachi, Pakistan

We collected the data in two approaches which is illustrated in the flowchart of patients inclusion (Fig. 1). First, telephonic data collection was done in the public hospital where we had the contact details of the patients who were brought to the emergency department. We used a standardized questionnaire to collect the data via telephone calls after acquiring verbal consent from the participants. A total of 126 patients were brought to the public tertiary hospital. We made 69 successful calls, while 38 contact details were not recorded during patient registration. Two cases refused to give consent, 8 cases were wrong contact numbers, and in 9 cases, the cellphones were off/not received after multiple calls at different times. Furthermore, more than 30% of the cases were contacted by two different research assistants two times and the collected information was compared for data validity and accuracy. The medical records and hospital files for chart review were not available in the public hospital.

In the second approach, we took basic demographic and key clinical variables from the hospital administration of the privately run trust hospital where the majority of the patients were taken to after the incident. The hospital recorded a total of 505 patients with a history of soybean dust exposure in the Keamari area. This hospital is located nearest to the incident site; therefore, the majority of the patients were shifted there. The contact details of these patients were not available to us for detailed interviewing and medical records were not maintained due to the high influx of patients more than the capacity of the hospital.

### Statistical analysis

We have used software SPSS version 19 for data entry and analysis. Descriptive statistics were reported to present frequencies and percentages for categorical variables. Mean ± standard deviation (SD) were calculated for continuous variables. Shapiro Wilk test was applied to check the normality of the quantitative variables.

## Results

A total of 574 patients were brought to the two hospitals in Karachi, Pakistan with a history of exposure to hazardous material from the Keamari area. The majority of the patients (*n* = 448, 78%) were presented to the nearby privately operated trust hospital. The remaining (*n* = 126, 22%) were taken to a public tertiary care center that is 10 km away from the incident area. The patients presented to these hospitals were without any emergency medical services support. Few patients were also taken to other public and private hospitals. However, we do not have the data from these sites, due to the lack of data collection permission and the complex processes of acquiring logistic and administrative support. The mean age of the victims was 32 years old with a standard deviation of 13.5 years. More than half of the patients (*n* = 319, 56%) were males. In terms of occupation, 29% (*n* = 20) were housewives, a quarter (26%, *n* = 26%) were salesperson, 22% (*n* = 16) were technical staff, and the remaining 23% (*n* = 16) were unemployed. In 28 cases (41%), the onset of symptoms occurred at home, in 27 cases (39%) the onset of symptoms started in the workplace and the remaining cases (*n* = 14, 20%) experienced the first symptoms while roaming around the roadside. Out of 69 cases that we called, 3 (4%) reported that some other family members also got ill and had allergic symptoms, while 66 (96%) reported that other family members were fine and did not experience any allergic symptoms (Table 1).

**Table 1:** Baseline & demographics characteristics of patients in HAZMAT incidence

Variable	*n* (%)
*Age in years*	
Mean (SD)	32 (± 13.5)
*Gender*	
Male	319 (56%)
Female	255 (44%)
Occupation*	
Housewife	20 (29%)
Salesperson	18 (26%)
Technical staff	15 (22%)
Unemployed	16 (23%)
Place of onset of symptoms*	
Home	28 (41%)
Workplace	27(39%)
On the way	14 (20%)
Other person affected at home*	
Yes	3 (4%)
No	66 (96%)
Other person affected at work*	
Yes	2 (3%)
No	65 (94.5%)
Unknown	2 (3%)

^*^^69 patients were called as contact numbers were available, for the rest of the patients, contact numbers and medical records were not available^

Out of a total of 69 patients, 6 (9%) reported that they are active smokers and 63 (91%) were non-smokers. Five patients (7%) had a known history of allergy, 45 (65%) reported that they are not allergic to any food or drug and the remaining 19 (28%) reported that their history of allergy is unknown. Only 1 patient reported a history of drug addiction. Nine patients (13%) reported that they have a chronic history of diseases, while 60 (87%) had no co-morbidity. The most common reported co-morbidity was a history of asthma (56%), followed by diabetes mellitus (22%) (Table 2).

**Table 2:** Allergic and past medical history characteristics of patients in HAZMAT incidence

Variable	*n* (%)
*Smoking status*	
Smoker	6 (9%)
Non-smoker	63 (91%)
*Allergic*	
Yes	5 (7%)
No	45 (65%)
Unknown	19 (28%)
*Drug addiction history*	
Yes	1 (1%)
No	68 (99%)
*Co-morbid*	
Yes	9 (13%)
No	60 (87%)
*Common co-morbid*^*#*^	
Asthma	5 (56%)
Diabetes	2 (22%)
Others	2 (22%)

^*^^Denominator is 9, the total patients reported having comorbid out of 69^

Most of the patients (*n* = 549, 96%) were discharged from the emergency department after initial management. A total of 16 cases (3%) were admitted to the in-hospital setting with 1- 2 days of the length of hospital stay. A total of 9 deaths (1.5%) were recorded in the privately operated trust hospital. The most common clinical manifestation was shortness of breath, reported in 94% of the cases, followed by neurological symptoms such as drowsiness, unconsciousness, or seizures experienced by 10% of the victims. Another 10% of the patients also reported gastrointestinal symptoms like nausea, vomiting, and abdominal pain. Only 1% of the patients reported irritation to the skin or eye (Table 3).

**Table 3:** Clinical outcome, sign, and symptoms of patients in HAZMAT incidence

Variable	*n* (%)
Disposition	
Discharged from ER	549 (96%)
Admitted	16 (3%)
Death	9 (1%)
Length of hospital Stay	
Range	1–2 days
Respiratory*	
Shortness of breath, cough,	
Yes	65 (94%)
No	4 (6%)
Skin*	
Irritation or rash	
Yes	1 (1%)
No	68 (99%)
Gastrointestinal*	
Nausea vomiting or abdominal pain*	
Yes	7 (10%)
No	62 (90%)
Neurological*	
Drowsiness or unconsciousness or seizure	
Yes	7 (10%)
No	62 (90%)

^*^^69 patients were called as contact numbers were available, for the rest of patients, contact numbers and medical records were not available^

## Discussion

In this study, the majority of the patients had unintentional soybean dust exposure at their home followed by workplace due to unloading of soybean at Karachi harbor. The respiratory tract was the most affected organ system followed by the gastrointestinal and neurological system. The majority of the patients got discharged from the emergency department. Sixteen patients were admitted, of whom nine were expired in this case series.

Soybean dust is generally caused by the unloading of soybean grains in bulk and can potentially trigger respiratory and other problems in people, particularly those who have pre-existing health conditions or exhibit sensitivity. Exposure to soybean dust has been implicated in causing various respiratory health symptoms. Numerous case studies have demonstrated the development of respiratory symptoms related to the loading or unloading of soya products [18–20]. Increases in emergency room visits for asthma-related symptoms following community soy exposure have been well documented [21, 22]. There have been various studies that showed a high prevalence of asthma-related symptoms in soybean factory workers [23–25]. Our study also demonstrated cough and shortness of breath to be the most common presenting symptom among exposed patients.

In this study, seven patients also developed vomiting and diarrhea due to soybean dust exposure. Soybeans are known to cause gastrointestinal (GI) symptoms, but this phenomenon has been profoundly reported in the pediatric population. GI symptoms are thought to be triggered by the non-IgE-mediated immunological response. However, the exact underlying mechanisms are still not well understood [11, 12]. Patients usually present with complaints of severe abdominal pain within one to four hours of exposure, followed by vomiting and diarrhea [13, 14]. The clinical condition resolves on its own and only requires supportive care. The data on the exact prevalence of GI symptoms because of soya bean dust exposure remains obscure.

Patients exposed to soybean dust in this study were presented to the privately run trust hospital and a public tertiary care hospital without field decontamination and Emergency Medical Services (EMS) support. At the time of the incident, the hospital physicians were unaware of the chemical nature of the incident and had difficulty in recognizing the chemical/ biological threats associated with the influx of patients affected by soybean dust. Failing to identify the chemical/ biological threats in a hospital environment can risk the safety of patients and healthcare professionals [26]. Early identification of hazardous material exposure is an important factor to initiate immediate action and prevent further risk of contamination [27]. The research team visited the emergency department of these hospitals where patients were taken after exposure to soybean dust. The team observed that these emergency departments had limited physical and human resources to manage chemically contaminated patients that can have potentially serious health consequences. There was no decontamination area in any hospital facility. This situation may not be different in most emergency care facilities in Pakistan. The hospital management of chemical incidents is complex and requires multifaceted response including alarm system to detect exposure to chemical, risk communication system, protocol to treat contaminated patients and decontamination facilities [28]. These measures will reduce secondary contamination of emergency care providers resulting from providing care to patients with exposure to hazardous materials [29]. In addition, emergency care providers must have a better understanding of toxicology to evaluate the potential health effects of hazmat exposure which is lacking in emergency care training in Pakistan [30].

Rapid response is essential to minimize the toxic effects of hazmat incidents [31]. Rapid response to a hazmat situation can minimize morbidity and mortality in those impacted while also limiting community exposure [32]. Decontamination in the field is the initial step in responding to hazmat events to ensure that toxic residues on a person or equipment are confined within the hot zone [33]. The overall goal of decontamination is to reduce the probability of harm to the affected person, prevent contamination of healthcare facilities, and therefore maintain the healthcare system’s function to continue delivering healthcare services [29, 34]. However, the decontamination process was poorly implemented by first responders in this hazmat incident, resulting in harmful exposure being transmitted to the affected person’s family.

### Recommendations

Despite of frequent occurrence of hazardous materials incidents in our country, we still lack well-equipped fully prepared emergency care facilities to handle patients contaminated with hazardous chemicals. Although some general guidelines exist, template protocols for the management of hazardous materials incidents are not readily available in every hospital. We encourage the establishment of emergency care facility centers, especially in the vicinity of industrial areas where designated and trained medical staff should be available for the treatment of exposed persons. We also recommend the development of a poison control center in the city that will be helpful in providing the information to individuals who have been exposed or are at risk of being exposed, assisting in triaging injured patients, and notifying the receiving health care facility. Furthermore, poison control centers also provides information on the toxicology of the chemicals involved in the incident and the management of exposed patients, and also assists in gathering data on exposures that may be useful for managing the incident or for surveillance [35, 36].

We strongly discouraged the presence of residential areas close to commercial industries. The government and relevant regulatory bodies should play their part in the proper transportation of these hazardous materials and educate the laborers and workers about the safe handling of such materials.

### Strengths and limitations

To the best of our knowledge, this is the first study from Pakistan that identified various gaps in the management of chemical disasters. We collected all the data on phone calls that could have resulted in missed information and recall bias. The data of deceased patients were also not available. Moreover, The sample size was also small, the study's outcomes might have been more robust had it been possible to reach a larger number of patients. We were unable to identify the exact concentration of soybean dust in air that caused symptoms in our patients. We could not collect the data on other factors that influence the exposure level of individual victims such as the location of the victims and the direction of the wind.

## Conclusion

An effective multidisciplinary emergency response system approach is needed to deal with such type of chemical emergencies in Pakistan. A multi-sectoral systematic approach is also required to address these incidents comprehensively including the trained and equipped pre-hospital system, integrated emergency medical response, and community-wide emergency response system.

## Data Availability

The data will be available at the reasonable request to Dr. Nadeem Ullah Khan, the corresponding author at nadeemullah.khan@aku.edu due to the Aga Khan University’s policy which can be viewed at aku.edu.

## Abbreviations

EMS: Emergency medical services
HAZMAT: Hazardous materials
ICCBS: International Center for Chemical and Biological Sciences
